# Redox Chemistry of Gold(I) Phosphine Thiolates: Sulfur-Based Oxidation

**DOI:** 10.1155/MBD.1994.419

**Published:** 1994

**Authors:** Tong Jiang, Gang Wei, Cristopher Turmel, Alice E. Bruce, Mitchell R. M. Bruce

**Affiliations:** 1 Department of Chemistry University of Maine Orono ME 04469-5706 USA

## Abstract

The redox chemistry of mononuclear and dinuclear gold(I) phosphine arylthiolate complexes was recently investigated by using electrochemical, chemical, and photochemical techniques. We now report the redox chemistry of dinuclear gold(I) phosphine complexes containing aliphatic dithiolate ligands. These molecules differ from previously studied gold(I) phosphine thiolate complexes in that they are cyclic and contain aliphatic thiolates. Cyclic voltammetry experiments of Au_2_ (LL)(pdt) [pdt  =  propanedithiol; LL = 1,2-bis(diphenylphosphino)-ethane (dppe), 1,3-bis(diphenylphosphino)propane (dppp), 1,4-bis(diphenylphosphino)butane (dppb), 1,5-bis(diphenylphosphino)pentane (dpppn)] in 0.1 M TBAH/CH_3_CN or CH_2_Cl_2_ solutions at 50 to 500 mV/sec using glassy carbon or platinum electrodes, show two irreversible anodic processes at ca. +0.6 and +1.1 V (vs. SCE). Bulk electrolyses at +0.9 V and +1.4 V result in n values of 0.95 and 3.7, respectively. Chemical oxidation of Au_2_(dppp)(pdt) using one equivalent of Br_2_ (2 oxidizing equivalents) yields 1,2-dithiolane and Au_2_(dppp)Br_2_. The reactivity seen upon mild oxidation ≤ +1.0 V is consistent with formal oxidation of a thiolate ligand, followed by a fast chemical reaction that results in cleavage of a second gold-sulfur bond. Oxidation at higher potentials (≥ +1.3 V) is consistent with oxidation of gold(I) to gold(III). Structural and electrochemical differences between gold(I) aromatic and aliphatic thiolate oxidation processes are discussed.


### REDOX CHEMISTRY OF GOLD(I) PHOSPHINE THIOLATES: SULFUR-BASED OXIDATION

Tong Jiang, Gang Wei, Cristopher Turmel, Alice E. Bruce and Mitchell R. M. Bruce*

Department of Chemistry, University of Maine, Orono, ME 04469-5706, USA

The redox chemistry of mononuclear and dinuclear gold(I) phosphine arylthiolate complexes was recently investigated by using electrochemical, chemical, and photochemical techniques. We now report the redox chemistry of dinuclear gold(I) phosphine complexes containing aliphatic dithiolate ligands. These molecules differ from previously studied gold(I) phosphine thiolate complexes in that they are cyclic and contain aliphatic thiolates. Cyclic voltammetry experiments of $Au_2$(LL)(pdt) [pdt = propanedithiol; LL = 1,2-bis(diphenylphosphino)-ethane (dppe), 1,3-bis(diphenylphosphino)propane (dppp), 1,4-bis(diphenylphosphino)butane (dppb), 1,5-bis(diphenylphosphino)pentane (dpppn)] in 0.1 M TBAH/$CH_3CN$ or $CH_2Cl_2$ solutions at 50 to 500 mV/sec using glassy carbon or platinum electrodes, show two irreversible anodic processes at ca. +0.6 and +1.1 V (vs. SCE). Bulk electrolyses at +0.9 V and +1.4 V result in n values of 0.95 and 3.7, respectively. Chemical oxidation of $Au_2$(dppp)(pdt) using one equivalent of $Br_2$ (2 oxidizing equivalents) yields 1,2-dithiolane and $Au_2$(dppp)$Br_2$. The reactivity seen upon mild oxidation $\leq$ +1.0 V is consistent with formal oxidation of a thiolate ligand, followed by a fast chemical reaction that results in cleavage of a second gold-sulfur bond. Oxidation at higher potentials ($\geq$ +1.3 V) is consistent with oxidation of gold(I) to gold(III). Structural and electrochemical differences between gold(I) aromatic and aliphatic thiolate oxidation processes are discussed.

### Introduction

The hypothesis that redox chemistry is important to the biochemistry of gold is appealing. Several models that include redox chemistry have been proposed to explain the biological activity of gold drugs.[1-4] For example, thiol/disulfide-rich membranes may 'shuttle' gold(I) through cells via a series of steps involving closely spaced thiol and disulfide sites.[2] Also, oxidation of gold(I) may be responsible for some of the serious side effects that accompany and limit the usefulness of gold drugs because gold(III) is known to be toxic.[1,3] Disulfides may also be a target site for gold(I) in cases where disulfide reduction is coupled to phosphine oxidation in a gold complex.[1,4]

Our group has been studying the electronic structure and reactivity of $d^{10}$ gold(I) complexes, especially gold(I) phosphine thiolate complexes.[5] These complexes are related to Auranofin, an orally active anti-arthritis drug containing triethylphosphine and tetraacetylthioglucose ligands.[6] Our previous investigations on the electronic structure of mononuclear and dinuclear gold(I) phosphine thiolate complexes suggest that the lowest energy transition is a ligand-to-metal charge transfer (S $\rightarrow$ Au) transition.[5a,b] We recently initiated a study on the redox reactivity of gold(I) phosphine complexes containing *p*-thiocresolate ligands.[5c] Cyclic voltammetry experiments on mononuclear and dinuclear gold(I) complexes show two irreversible anodic processes near +0.6 and +1.5 V. Bulk electrolyses experiments were carried out at +1.0V and +1.5V to measure the number of electrons transferred (n) for each oxidation process. For mononuclear gold(I) complexes the n values are 0.5 (at +1.0V) and 2 (at +1.5V), while dinuclear complexes have n values of 1 (at +1.0V) and 5 (at +1.5V). Thus, in the first oxidation process, only one electron is transferred per two Au, P, or S sites. Furthermore, during electrolysis, *p*-tolyl disulfide [$(p\text{-tc})_2$] forms. Chemical oxidation as well as photolysis also yield $(p\text{-tc})_2$. The n values and chemical reactivity seen upon mild electrochemical oxidation are consistent with formal oxidation of one thiolate ligand, followed by a fast chemical reaction that results in formation of a sulfur-sulfur bond.

Table I includes data on the oxidation of gold(I) or gold(II) complexes bound to sulfur ligands. Cyclic thioethers, such as [15]ane$S_5$ form gold(I) complexes which undergo oxidation at low

**Table I.** Oxidation Potentials for Selected Gold Complexes and Ligands in Nonaqueous Media.

| Formula | Structure | $E_{ox}$ | Assignment | Ref. |
|---|---|---|---|---|
| $[Au([15]aneS_5)][PF_6]$ |  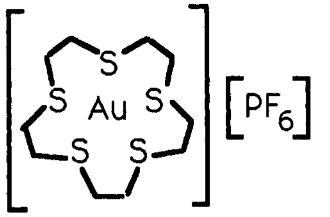 | +0.12, +0.46 V[a] vs. $Cp_2Fe/Cp_2Fe^+$ | Au(I)/Au(II) and Au(II)/Au(III) | 7 |
| $\{Au(abt)_2\}_2$ |  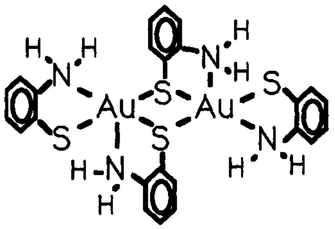 | +0.26 V, +0.59 V[b] vs. SCE | Au(II)/Au(III) | 8 |
| $[Au(CH_2)_2PPh_2]_2$ |  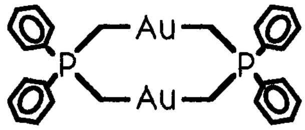 | +0.11 V, +0.235 V[c] vs. Ag/AgCl | Au(I)/Au(II) | 9 |
| $[Au(dppe)_2][PF_6]$ |  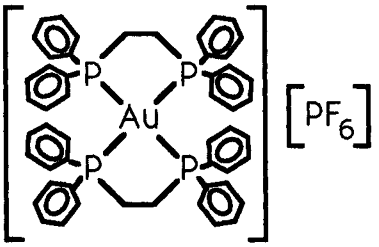 | +0.458 V[a] vs. S.C.E. | Au(I)/Au(III) | 10 |
| $Au(PPh_3)Cl$ |  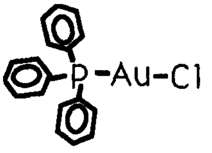 | +1.5 V[d] vs. $Cp_2Fe/Cp_2Fe^+$ | Au(I)/Au(III) | 11 |
| | | +1.6 V[e] vs. Ag/AgCl | Phosphine based | 12 |
| $PPh_3$ |  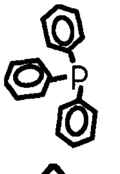 | +0.12 V[f] vs. SCE | Phosphine based | 13 |
| naptho[1,2-*b*,*c*]-1,5-dithiocin |  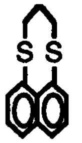 | +0.47 V[f] vs. $Ag/AgNO_3$ | Sulfur based | 14 |

[a]0.1 M $TBAH/CH_3CN$. [b]0.1M $TEAP/CH_3CN$. [c].1M TBAH/THF. [d]$TBAP/CH_3CN$. [e]$TEAB/CH_3CN$. [f]0.1M $LiClO_4/CH_3CN$.

positive potentials.[7] For example, the redox couples of $[Au([15]aneS_5)]^+$ are reversible and occur at +0.12 and +0.46 V vs. ferrocene/ferrocinium. The redox couples have been assigned as $Au^{I/II}$ and $Au^{II/III}$, respectively. The bis-gold thiolate complex $\{Au(abt)_2\}_2$ (abt = *o*-aminobenzenethiolate) which contains two gold(II) ions coordinated to sulfur and nitrogen donor ligands, undergoes reversible redox processes at +0.26 and +0.59 V vs. SCE that have been assigned as $Au^{II/III}$ redox couples.[8]

Electrochemical studies of gold phosphous complexes are also available. For example, the bis-gold phosphous ylide complex, $Au_2[(CH_2)_2PPh_2]_2$, undergoes oxidation at mild potentials.[9] Cyclic voltammetry experiments show two quasi-reversible redox processes at +0.11 V, and +0.24 V (vs. Ag/AgCl). These molecules readily undergo oxidative addition reactions with alkyl halides to form $Au^{II}$-$Au^{II}$ complexes. A molecular orbital calculation identifies the HOMO orbital in $[Au(CH_2)_2PPh_2]_2(CH_3)Br$ as metal-metal bonding.[9] On this basis, it is reasonable to suggest that the redox couple involves $Au^{I/II}$. Another example is the oxidation of molecules such as $[Au(dppe)_2]^+$ which also undergoes oxidation at mild potentials ($E_{1/2}$=+0.46 V vs. SCE).[10] On the basis of the peak-to-peak separations measured by cyclic voltammetry and *n* values from bulk electrolyses experiments, the redox processes were assigned as involving the $Au^{I/III}$ couple. Anderson, et al. have reported on the oxidation of $AuPPh_3Cl$, which undergoes oxidation at ca. +1.5 V vs. the ferrocene/ferrocinium couple.[11] The oxidation is assigned as involving the $Au^{I/III}$ couple on the basis of electrochemical and spectroelectrochemical studies. Recently, this assignment has been challenged by Rakhimov, et al. who suggest that oxidation of $AuPPh_3Cl$ (ca. +1.6 V vs. Ag/AgCl) involves phosphine.[12] In addition, the ligands themselves may undergo oxidation at low positive potentials. This is evident by the oxidation of $PPh_3$ (+0.12V vs. SCE)[13] or naptho[1,2-*b*,*c*]-1,5-dithiocin (+0.47 V vs. Ag/0.1 M $AgNO_3$).[14] Finally, it is important to note that free thiolate anions, such a *p*-thiocresolate, oxidize near 0 V (vs. SCE),[15] and that the thiol, *p*-thiocresol, oxidizes near +1.5 V.[16] These electrochemical studies demonstrate that gold, phosphine, and sulfur ligands may be redox sites. Therefore, at issue are the following questions concerning the oxidative behavior of gold(I) phosphine thiolate complexes: (1) which of the three potential redox sites, gold(I), phosphine, or thiolate are redox active, (2) do significant following reactions occur after oxidation, and (3) do any of these sites undergo redox chemistry at low enough potentials to be biologically accessible? As a continuation of our studies on gold phosphine thiolate complexes, we now report electrochemical and chemical oxidation studies of dinuclear gold(I) complexes containing aliphatic, dithiolate ligands.

### Materials and Methods

**Materials.** Acetonitrile (Burdick & Jackson UV grade), and methylene chloride (spectrophotometric grade, Aldrich) for the electrochemical experiments were used as received. Deuterated solvents were purchased from Cambridge Isotope Laboratories. The supporting electrolyte, tetra-N-butylammonium hexafluorophosphate (TBAH), was prepared by metathesis of tetra-N-butylammonium bromide (TBABr), and $HPF_6$ in water.[17] TBAH was purified by recrystallization from methylene chloride/ether and dried at 80 °C under vacuum for more than 24 hours. The supporting electrolyte, potassium hexafluorophosphate (98%, Aldrich) was used as received. The gold complexes were prepared according to previously published methods.[5a] $HAuCl_4 \cdot 3H_2O$ was purchased from Aldrich or Aithaca Chemical; phosphine ligands were purchased from Strem or Aldrich; and *p*-thiocresol and *p*-tolyldisulfide were purchased from Aldrich. $^1H$ NMR spectra were recorded on Varian 200 MHz or 300 MHz spectrometers at ambient temperature.

**Abbreviations.** The following abbreviations are used: *p*-tc = *p*-thiocresol; LL = 1,2-bis(diphenylphosphino)ethane (dppe), 1,3-bis(diphenylphosphino)propane (dppp),

1,4-bis(diphenyl-phosphino)butane (dppb), 1,5-bis(diphenylphosphino)pentane (dpppn).

**Cyclic voltammetry (CV) experiments.** CV experiments were conducted by using an EG&G Princeton Applied Research 273 potentiostat/galvanostat. A remote computer controller and the program HEADSTRT (EG&G PAR) were used to acquire and store data from the PAR 273. CV data were then converted to SPECTRA CALC (Galactic Software) format using a series of conversion programs.

CV measurements were performed in acetonitrile or methylene chloride with 0.1 M TBAH as supporting electrolyte. Fresh solutions containing electrolyte (5 mL) were prepared prior to each CV experiment. Each solution was deoxygenated by purging with nitrogen for 5 minutes. Background CV's were acquired before the addition of gold compound. A platinum (1.6 mm diameter) or glassy carbon (3.0 mm) disk working electrode, a platinum wire auxiliary electrode, and a saturated potassium calomel reference electrode (S.C.E.) comprised the three-electrode system. The working electrode was polished before each set of experiments with diamond polish (Metadi) and was wiped clean prior to each measurement. The auxiliary electrode was lightly sanded before each set of experiments with fine sand paper. Potentials are reported vs. S.C.E. at room temperature and are not corrected for junction potentials. In general, all the experiments were repeated several times.

**Controlled potential electrolysis experiments.** The electrolysis experiments were performed using an EG&G Princeton Applied Research 273 potentiostat/galvanostat set in the potentiostat mode and operated at the specified potential. A three compartment cell designed for handling air sensitive materials was used for all electrolysis experiments.[18] The reference electrode was S.C.E. brought into the main compartment via a Luggin capillary. The main compartment contained a cylindrical platinum mesh working electrode and a Teflon stir bar centered within the platinum mesh. The auxiliary platinum mesh electrode was separated from the main compartment by a fine glass frit.

In each constant potential electrolysis experiment, the apparatus was assembled, after oven drying, and cooled under an atmosphere of nitrogen. The electrolysis solution (0.1 M TBAH) was introduced into the cell and was stirred and degassed with nitrogen for a minimum of ten minutes. The potentiostat was set to the potential of interest, the cell turned on, and after the current had reached a steady state, typically within 5 minutes, the background current ($i_{bkg}$) was measured and recorded. The potential and internal coulometer of the PAR 273 was then reset to zero. Gold compound (25 to 46 mg, ≈ 0.03-0.05 mmol) was added to the cell, and the solution was degassed with nitrogen for an additional ten minutes. The same potential used to determine the background current was then applied to the solution. During the electrolysis oxidation experiment, the total current ($_{total}$) was measured as a function of time. The rate of stirring remained approximately constant throughout the experiment. When $i_{total} \approx i_{bkg}$, the assumption was made that electrolysis was complete and the experiment was stopped. The number of electron equivalents (n) passed during the experiment was calculated using Faraday's Law and by assuming that $i_{bkg}$ was a constant during the electrolysis experiment. Thus, the total number of coulombs passed during the oxidation of a gold(I) complex ($Q_{ox}$) was calculated by subtraction of the coulombs from the background process ($Q_{bkg} = i_{bkg}$ x time) from the total number of coulombs using $Q_{total} - Q_{bkg} = Q_{ox}$. Constant potential electrolysis of ferrocene at +1.5 V vs. SCE, used to calibrate the cell, gave an *n* value of 0.93 (expected *n* = 1.0).

**Chemical oxidation experiment.** 15.0 mg of $Au_2$(dppp)(pdt) (1.76 x $10^{-5}$ mol) was dissolved in 3 mL $CD_2Cl_2$, under $N_2$. A 0.6 mL aliquot of a 0.03 M $Br_2/CD_2Cl_2$ solution (1.8 x $10^{-5}$ mol $Br_2$) was added and the reaction mixture was stirred in an ice bath for 1 hr, during which time the red-brown color of $Br_2$ disappeared. An aliquot of the reaction mixture was transferred to an NMR tube.

$^1H$ NMR ($CD_2Cl_2$): δ (ppm ) = 7.8-7.4 (m, 20H, phenyl), 3.1 (t, 4H, $SCH_2CH_2$), 2.8 (m, 4H, $PCH_2CH_2$), 2.25 (quint., 2H, $SCH_2CH_2$), 1.9 (m, 2H, $PCH_2CH_2$).

### Results

**Cyclic Voltammetry.** Oxidative cyclic voltammetry experiments on $Au_2$(LL)(pdt) (LL = dppe, dpppn) were performed at Pt and glassy carbon electrodes using either 0.1 M TBAH/$CH_3CN$ or $CH_2Cl_2$ solutions (Table II). Cyclic voltammetry experiments were performed at scan rates between 50 and 500 mV/sec and several replications were obtained at each scan rate. The switching potential was also varied in order to characterize different oxidation processes. Adsorption of gold complexes to the electrode surface occurred at all electrode/solvent combinations investigated and was minimized by wiping the electrode between each CV scan. In general, scan rates were randomly varied using tables of random permutations in order to minimize any sequential effects of adsorption.[19]

Oxidation of $Au_2$(dppe)(pdt) at glassy carbon in $CH_3CN$ (ca. 0.5 mM) shows a broad anodic wave with peak potential, $E_{p1}{}^A$, at +0.6 V vs. SCE (Table II, Figure 1). There is no return component of this oxidation process, i.e. it is irreversible. The broadness of the wave can be quantified by taking the difference between the potential at maximum current ($E_{p1}{}^A$) and the potential at half-current ($E_{p1/2}{}^A$). This difference is ca. 150 mV for the scan shown in Fig 1B. (For comparison, a value of 59 mV is predicted for a reversible one electron process.) The corresponding set of oxidative CV experiments of $Au_2$(dpppn)(pdt) show similar broad oxidation processes except that the peak near +0.6 V is sometimes very weak (see Table II).

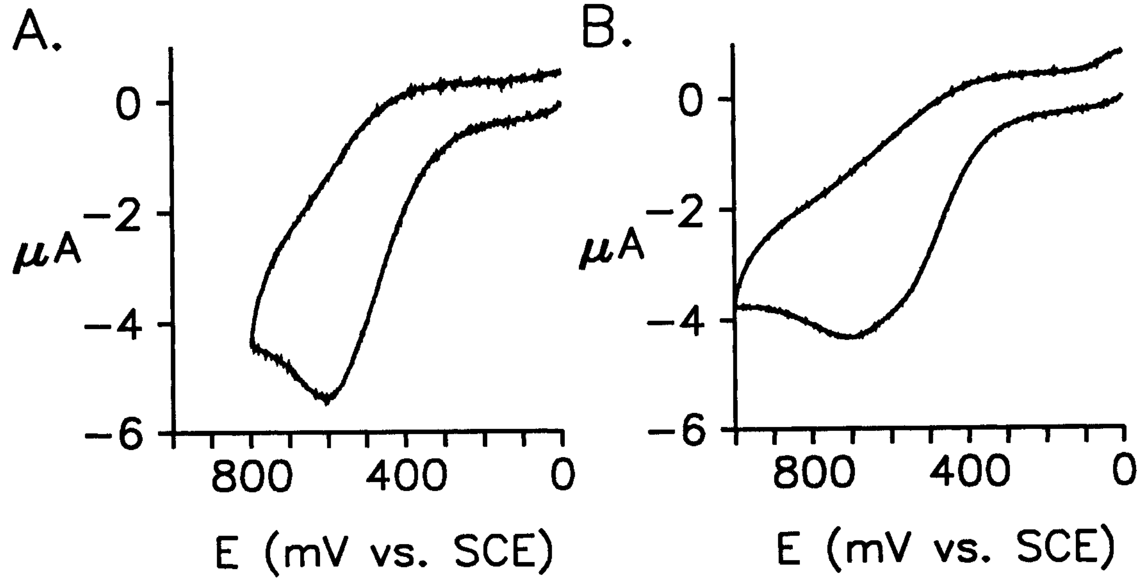

**Figure 1.** Cyclic Voltammetry of $Au_2$(dppe)(pdt) at a glassy carbon electrode; scan rate 50 mV/s. A) 0.47 mM, 0.1 M TBAH/$CH_3CN$, 0 to +0.8V (vs. SCE). B) 0.48 mM, 0.1 M TBAH/$CH_2Cl_2$, 0 to +1.0V.

Changing the switching potential to +2.0 V shows a second, irreversible oxidation process at ca. +1.11V (Figure 2, glassy carbon electrode). The sharpness of the second oxidation wave,

$E_{p1}^{A}$ - $E_{p1/2}^{A}$ = 60 mV, and the increase in current relative to the first oxidation wave, suggest that the second oxidation process involves more electrons than the first process.

Results of oxidative cyclic voltammetry experiments on mononuclear and dinuclear *p*-thiocresolate gold(I) complexes are shown in Table II for comparison. It is evident that when the results for each complex under various electrode/solvent combinations are reviewed, the potentials and waveshapes for the first oxidation process are similar for all complexes studied. In contrast, the second oxidation process occurs at significantly lower potentials (less positive) in the pdt complexes than the *p*-tc complexes.

**Constant Potential Electrolysis.** Bulk electrolysis of $Au_2$(LL)(pdt) (LL = dppp, dppb), at +0.9V using a Pt working mesh electrode in 0.1 M TBAH/$CH_3CN$ solution results in n = 1.0. Thus, the first oxidation process appears to involve *one* electron per *two* sulfur, gold, or phosphorus redox sites. This tends to preclude a 'simple' mechanism involving complete oxidation at any one redox site, e.g. at all sulfur sites. There is a possibility that adsorption occurs, effectively passivating the electrode. However, removing the bulk electrolysis solution after electrolysis of $Au_2$(LL)(pdt) *at +0.9V* and performing cyclic voltammetry experiments, shows no oxidation waves below +1.0 V, yet an oxidation wave at ca. +1.1 V is still observed. Similar results are observed for cyclic voltammetry experiments on electrolysis solutions of $Au_2$(LL)(*p*-tc) complexes except that the second oxidation wave occurs at ca. +1.5 V. These results suggest that the oxidation process below +1.0V in the cyclic pdt complexes is complete. Further experiments are in progress to rule out the possibility that adsorption at the new working (CV) electrode may obscure the first wave. The bulk electrolyses results for the first oxidation process are consistent with an EC mechanism in which a fast following reaction occurs.

**Chemical Oxidation.** The formal oxidation potential of bromine, +0.82 V vs. S.C.E.,[20] makes it suitable as a one-electron oxidant for the gold(I) phosphine thiolate complexes. Furthermore, the reduced species of bromine, $Br^-$, is a good ligand for gold. When one equivalent of $Br_2$ (two oxidizing equivalents) is added to $Au_2$(dppp)(pdt) and the solution is monitored by $^1H$ NMR, the peaks associated with the starting complex quickly disappear and 1,2-dithiolane forms quantitatively. Figure 3A shows the spectrum of $Au_2$(dppp)(pdt) before addition of $Br_2$. After addition of one equivalent of $Br_2$, the starting gold complex has disappeared and new peaks at 3.1 (triplet) and 2.25 (quintent) ppm due to 1,2-dithiolane[21] have appeared (Figure 3B). The chemical shifts and appearance of the remaining peaks in Figure 3B are consistent with coordinated phosphine and are assigned as $Au_2(dppp)(Br)_2$ by comparison to an authentic sample of $Au_2(dppp)(Cl)_2$ (see Figure 3C).

### Discussion

**First Oxidative Process.** Previous electrochemical, chemical, and photochemical studies on mononuclear and dinuclear gold(I) complexes containing *p*-thiocresolate ligands can be summarized by Scheme I.[5c] Mild electrochemical oxidation (<+1.0 V vs. SCE) appears to lead to an irreversible one-electron oxidation with formation of the disulfide, $(p\text{-tc})_2$. On the basis of scan rate dependent studies and bulk electrolysis experiments, an EC mechanism was assigned for the first oxidation process.[22] Photolysis ($\lambda$ > 330 nm) and chemical oxidation by $Br_2$ also result in formation of $(p\text{-tc})_2$.

The results of electrochemical and chemical oxidation experiments on cyclic, dinuclear gold(I) complexes are summarized in Scheme II. Electrochemical oxidation at <+1.0 V vs. SCE also appears to lead to an irreversible one-electron oxidation process. Oxidation potentials, wave shapes, and n values in the cyclic, propanedithiolate gold(I) complexes are very similar to the *p*-thiocresolate gold(I) complexes. The lowest energy electronic transition in both series of complexes was previously assigned as a S$\rightarrow$Au charge transfer.[5a,b] On the basis of these

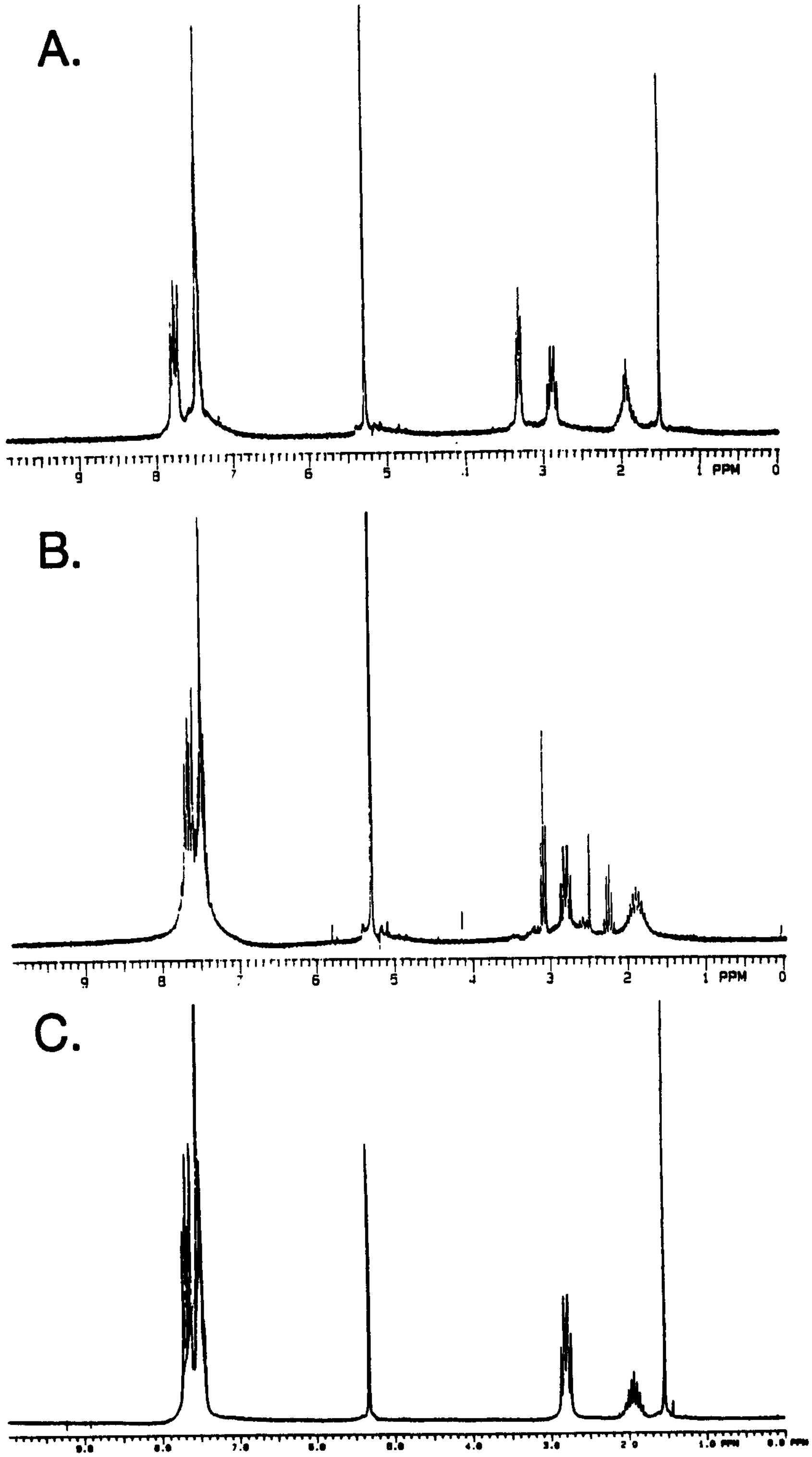

**Figure 3.** $^{1}H$ NMR spectra in $CD_2Cl_2$ of the reaction of $Au_2(dppp)(pdt)$ and $Br_2$. A) Before addition of $Br_2$ B) After addition of 1 equivalent of $Br_2$. C) $^{1}H$ NMR spectrum of $Au_2(dppp)Cl_2$ in $CD_2Cl_2$.

Scheme I

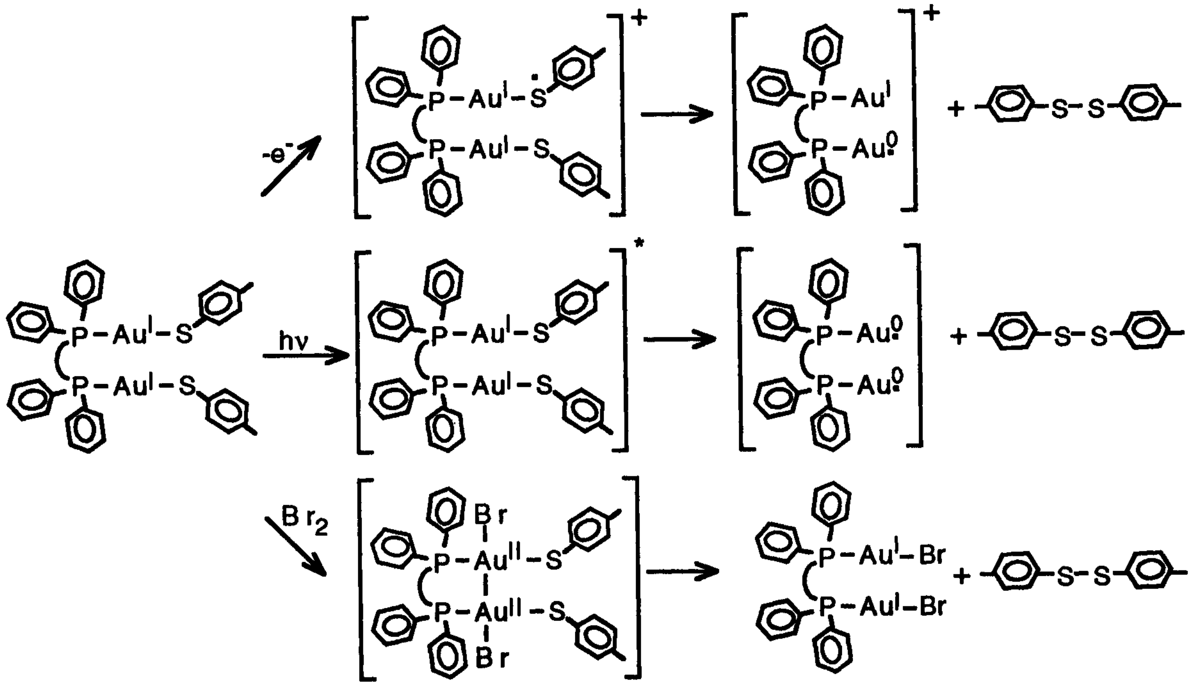

Scheme II

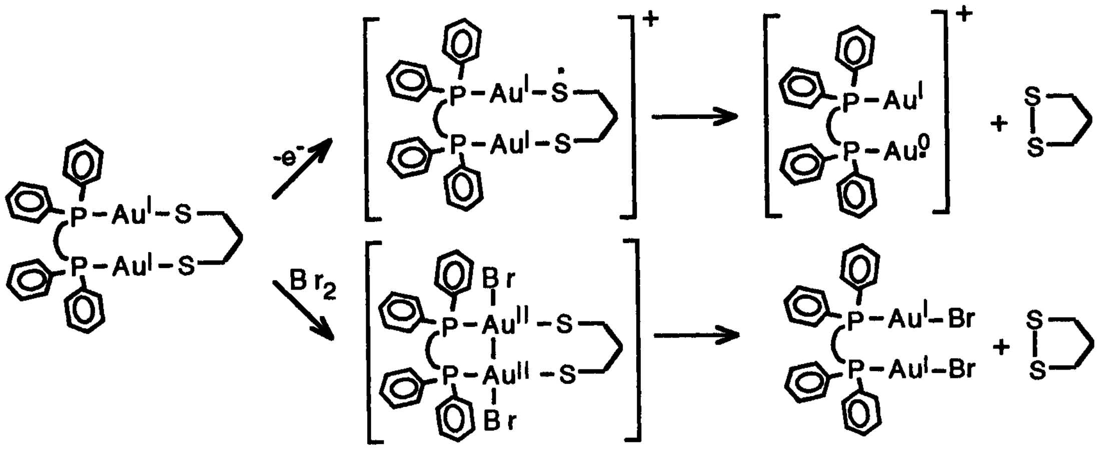

similarities, we propose that mild electrochemical oxidation (<+1.0 V) of the cyclic gold(I) complexes results in oxidation of the propanedithiolate ligand, followed by a rapid chemical step that leads to gold-sulfur bond cleavage and formation of the cyclic disulfide, 1,2-dithiolane; i.e. an EC mechanism.

The chemical oxidation of $Au_2$(dppp)(pdt) by $Br_2$ suggests an oxidative addition/reductive elimination mechanism. 1,2-Dithiolane has been identified by $^1H$ NMR spectroscopy as a product in the reaction of $Au_2$(dppp)(pdt) and one equivalent $Br_2$ (two oxidizing equivalents). In this case, the gold product has been identified as the dinuclear gold(I) complex, $Au_2(dppp)Br_2$. Previous studies by Fackler, et al. and Schmidbaur, et al. show that halogens oxidatively add to cyclic, dinuclear gold(I) complexes with strong σ-donor ligands to form Au(II)/(II) or Au(I)/(III) products, where the stability depends on the nature of the ligands.[9,23] It is plausible therefore to propose that oxidative addition of $Br_2$ to $Au_2$(dppp)(pdt) gives a Au(II)/(II) dinuclear complex which rapidly reductively eliminates 1,2-dithiolane.

**Second Oxidative Process.** Comparison of the cyclic voltammograms of solutions of $Au_2$(dppp)(*p*-tc)$_2$ and $Au_2$(dppp)(pdt) result in an interesting observation. Cyclic voltammograms show that the second oxidation wave of $Au_2$(dppp)(pdt) occurs at significantly lower potentials than the second oxidation wave of $Au_2$(dppp)(*p*-tc)$_2$. This observation suggests that the structures of the electrochemically active intermediates, generated after the first oxidation process for each complex, are different, because on the CV timescale (e.g. 50 mV/s), several seconds elapse between the first and second oxidation processes. If both sulfur ligands were rapidly lost after the first oxidation processes for $Au_2$(dppp)(*p*-tc)$_2$ and $Au_2$(dppp)(pdt), the same gold intermediate, e.g. $Au_2(dppp)^+$, would be formed. Thus, this result supports the idea that only one sulfur redox center is electrochemically oxidized.

Bulk electrolysis experiments on $Au_2$(LL)(pdt) (LL = dppp, dppb) at ≥ +1.3 V result in n = 3.5 and 4.2, respectively, consistent with an electrochemical oxidation involving generation of disulfide followed by oxidation of gold to Au(III). At the beginning of the experiments, the solutions are cloudy, due to the low solubility of the complexes in $CH_3CN$. However, during electrolyses, the solutions clear and then become yellow. Cyclic voltammetry experiments conducted after the bulk electrolyses were complete, show waves which previously have been ascribed to the reduction of $Au^{III}$ in solution.[11] The products of the second oxidation process are unknown at this time.

**Conclusion.** Savéant and coworkers recently reported electrochemical studies of thiophenoxide and para-substituted thiophenoxide anions.[15] At scan rates of 0.025 to 10 V/s, the cyclic voltammograms are irreversible. However, at higher scan rates, e.g. 1,000 V/sec, and low thiolate concentration, partial reversibility is observed for some of the compounds. This behavior is attributed to a fast electron transfer step followed by a very fast dimerization to form disulfide. For (*p*-tc)$^-$, the formal electrochemical oxidation potential is reported as +0.04 V (vs. SCE).[15]

The overall effect of a fast following chemical step is to decrease the observed oxidation potential compared to the formal oxidation potential, $E^\circ$.[15] Thus, a molecule appears easier to oxidize than it does in the absence of any chemical step. These observations allow us to speculate that the formal oxidation potentials of mono- and dinuclear gold(I) phosphine thiolate complexes are ≥ +0.56 V. *Thus, coordination of thiolates to gold(I) phosphines shifts the oxidation of a thiolate by >+500 mV.* An increase in ligand oxidation potential upon coordination to a transition metal is consistent with the central field effect as recently discussed by Dodsworth, Vlcek, and Lever.[24] This result may have biological implications in as much as coordination of gold(I) to thiolates in vivo may serve to protect high-affinity thiols from oxidation and thereby modify certain thiol/disulfide equilibria in cells. Alternatively, oxidation of a phosphine gold(I) moiety attached to cysteine within a protein may lead to formation of disulfide bridges that could dramatically alter the structure of a protein. Furthermore, on the basis of our electrochemical results, oxidation of gold(I) phosphine thiolates to gold(III) would require a more oxidizing environment than is typically found in cells.[25] More powerful oxidants capable of oxidizing gold(I), such as $H_2O_2$ and HOCl, are released by leukocytes as a consequence of an immune response.[26]

Finally, it is interesting to note the similarity in reactivity of $d^0$ and $d^{10}$ metal thiolates. We have shown that electrochemical oxidation (+1.0 V vs. SCE), chemical oxidation, and photolysis of gold(I) phosphine thiolate complexes leads to formal oxidation of the thiolate ligands, cleavage of gold-sulfur bonds, and formation of disulfide. Metal-sulfur bond cleavage is also observed upon photolysis or oxidation of Ti(IV) thiolates. For example, PhSSPh forms when $Cp_2Ti(SPh)_2$ is either irradiated at $\lambda$>530 nm[27] or oxidized by $Br_2$.[28] We are continuing to investigate the factors that influence the redox chemistry of gold(I) thiolates in an effort to better understand the biochemistry of gold phosphine thiolate complexes.

**Acknowledgment**. The authors gratefully acknowledge the National Institutes of Health (AR39858) and the University of Maine BRSG funds for financial support.

**References**

1) For reviews on the use of gold complexes in the treatment of rheumatoid arthritis see: (a) Smith, W. E.; Reglinski, J. in *Perspectives on Bioinorganic Chemistry*, Hay, R. W.; Dilworth, J. R.; Nolan, K. B., Eds.; Jai Press Ltd: London, 1991, 183. (b) Sadler, P. J. in *Adv. in Inorg. Chem.*,1991, **36**, 1. (c) Walz, D. T. *Adv. in Inflammation Research*, Otterness, I.; Capetola, R.; Wong, S., Eds.; Raven Press: New York, 1984, **7**, 239. (d) Brown, D. H.; Smith, W. E. *Chem. Soc. Rev.* 1980, **8**, 217. (e) Shaw, C. F. III *Inorganic Perspectives in Biology and Medicine*, 1979, **2**, 287.

(2) Snyder, R. M.; Mirabelli, C. K.; Crooke, S. T. *Biochem. Pharmacology*, 1986, **35**, 923.

(3) (a) Isab, A. A.; Sadler, P. J. *Biochem. Biophys. Acta*,1979, **492**, 322. (b) Shaw, C. F., III; Cancro, M. P.; Witkiewicz, P. L.; Eldridge, J. E. *Inorg. Chem.*, 1980, **19**, 3198.

(4) (a) Malik, N. A.; Otiko, F.; Sadler, P. J. *J. Inorg. Biochem.*, 1980, **12**, 317. ( b) Razi, M. T.; Otiko, G.; Sadler, P. J. *ACS Symp. Ser.*, 1983, **209**, 371.

(5) (a) Narayanswamy, R; Young, M. A.; Parkhurst, E.; Ouellette, M.; Kerr, M. E.; Ho, D. M.; Elder, R. C.; Bruce, A. E.; Bruce, M. R. M. *Inorg. Chem.*, 1993, **32**, 2202. (b) Jones, W. B., Yuan, J.; Narayanaswamy, R.; Young, M. A.; Elder, R. C.; Bruce, A. E.; Bruce, M. R. M. *Inorg. Chem.*, accepted for publication. (c) Turmel, C.; Jiang, T.; Wei, G.; Morrison, R.; Narayanaswamy, R.; Bruce, A. E.; Bruce, M. R. M. *J. Am. Chem. Soc.* (submitted). (d) Foley, J.; Fort, R. C. Jr.; McDougal, K.; Bruce, M. R. M.; Bruce, A. E. *Metal-Based Drugs*, accepted for publication.

(6) (a) Sutton, B. M.; McGusty, E.; Walz, D. T.; DiMartino, M. J. *J. Med. Chem.* 1972, **15**, 1095. (b) Hill, D. T.; Sutton, B. M. *Cryst. Struct. Commun.*,1980, **9**, 679.

(7) Blake, A. J.; Taylor, A.; Schröder, M *J. Chem. Soc., Chem. Commun.*, 1993, 1092.

(8) The oxidation state of each gold has been assigned as +2 on the basis of ESR data. Koley, A. P.; Purohit, S.; Ghosh, S.; Prasad, L. S.; Manoharan, P. T. *J. Chem. Soc. Dalton Trans.*, 1988, 2607.

(9) Basil, J. D.; Murray, H. H.; Fackler, J. P. Jr.; Tocher, J.; Mazany, A. M.; Trzcinska-Bancroft, B.; Knachel, H.; Dudis, D.; Delord, T.J.; Marler, D. O. *J. Am. Chem. Soc.*,1985, **107**, 6908.

(10) McArdle, J. V.; Bossard, G. E. *J. Chem. Soc. Dalton Trans.*, 1990, 2219.

(11) Anderson, J. E.; Sawtelle, S. M.; McAndrews, C. E. *Inorg. Chem.*, 1990, **29**, 2617.

(12) Rakhimov, R. D.; Butin, K. P.; Grandberg, K. I. *J. Organomet. Chem.*,1994, **464**, 253.

(13) Bard, A. J. *Encyclopedia of Electrochemistry of the Elements*, Vol. III; Marcel Dekker, Inc.: New York, 1975, p. 21.

(14) Glass, R. S.; Andruski, S. W.; Broeker, J. L.; Firouzabadi, H.; Steffen, L. K.; Wilson, G. S. *J. Am. Chem. Soc.*, 1989, **111**, 4036.
(15) Andrieux, C. P.; Hapiot, P.; Pinson, J.; Savéant, J-M. *J. Am. Chem. Soc.*,1993, **115**, 7783.
(16) Jiang, T. *Electrochemical Studies of Gold(I) Phosphine Complexes*, University of Maine, M.S. thesis, 1991.
(17) Bruce, M. R. M.; Megehee, E.; Sullivan, B. P.; Thorp, H.H., O'Toole, T. R.; Downard, A.; Pugh, J. R.; Meyer, T. J. *Inorg. Chem.*, 1992, **31**, 4864.
(18) Bolinger, C. M.; Story, N; Sullivan, B. P.; Meyer, T. J. *Inorg. Chem.*, 1988, **27**, 4582.
(19) Cochran, W. G.; Cox, G. M. *Experimental Design*, 2nd ed.; John Wiley & Sons, New York, 1957; pp 577-595.
(20) Lide, D. R. *Handbook of Chemistry and Physics*, 71st ed.; CRC Press: Florida, 1990, 8-17.
(21) Green, M.; Lown, E. M.; Strausz *J. Am. Chem. Soc.*, 1984, **106**, 6938.
(22) Greef, R.; Peat, R.; Peter, L. M.; Pletcher, D.; Robinson, J. *Instrumental Methods in Electrochemistry*, Ellis Horwood, Ltd: Chichester, 1985.
(23) See for example (a) Schmidbaur, H.; Mandl, J. R.; Frank, A.; Huttner, G. *Chem. Ber.*, 1976, **109**, 466. (b) Schmidbaur, H.; Hartman, C.; Reber, J.; Müller, G. *Angew. Chem. Int. Ed. Engl.*, 1987, **26**, 1146. (c) Mazany, A. M.; Fackler, J. P., Jr. *J. Am. Chem. Soc.*, 1984, **106**, 801. (f) Khan, M. N. I. Wang, S.; Fackler, J. P., Jr. *Inorg. Chem.*, 1989, **28**, 3579 and references therein. (g) Vicente, J.; Chicote, M.-T.; Saura-Llamas, I. *J. Chem. Soc., Dalton Trans.*, 1990, 1941.
(24) Dodsworth, E. S.; Vlcek, A. A.; Lever, A. B. P. *Inorg. Chem.*, 1994, **33**, 1045.
(25) Biologically accessible redox potentials generally fall between -0.5 - 0 V. Frausto da Silva, J. J. R.; Williams, R. J. P. *The Biological Chemistry of the Elements*, Clarendon Press: Oxford, 1991, 138.
(26) (a) Reglinski, J.; Paterson, D. E.; Latimer, S.; Campbell, J. M.; Wilson, R.; Sturrock, R. D.; Smith, W. E. "Myocrisin Mediated Oxidative Stress", 3rd International Conference on Gold and Silver in Medicine, Milwaukee, MN, August, 1994. (b) Shaw, C. F., III; Schraa, S.; Gleichmann, E.; Grover, Y. P.; Dunemann, L.; Jagarlamundi, A. "Redox Chemistry and Cyanide in the Formation of Gold Metabolites", 3rd International Conference on Gold and Silver in Medicine, Milwaukee, MN, August, 1994.
(27) Bruce, A. E.; Bruce, M. R. M.; Tyler, D. R. *J. Am. Chem. Soc.*, 1984, **106**, 6660.
(28) Shaver, A.; Morris, S.; Desjardins, A. *Inorg. Chim. Acta*, 1989, **161**, 11.

